# Childhood Trauma and Disordered Eating Attitudes and Behaviours in Adulthood: Investigating the Role of Daily Stress and Daily Perfectionistic Thinking

**DOI:** 10.1007/s40653-026-00834-1

**Published:** 2026-02-25

**Authors:** Amy Willgoose, Daryl B. O’Connor

**Affiliations:** 1 Leeds Institute of Health Sciences, School of Medicine, University of Leeds, Leeds, UK; 2 School of Psychology, University of Leeds, Leeds, UK

**Keywords:** Early life adversity, Adverse childhood experiences, Daily hassles, snacking

## Abstract

Background: Childhood trauma has been linked to diagnosed eating disorders and disordered eating attitudes and behaviours, however few studies have investigated these relationships at the daily level in non-clinical populations. Furthermore, stress and perfectionistic thinking have been implicated separately within the childhood trauma and disordered eating literature, but no research has examined these at the daily level in conjunction with childhood trauma. The present study aimed to investigate the relationship between childhood trauma and daily disordered eating measures whilst also considering the role of daily stress and perfectionistic thinking. Methods: Participants completed a background questionnaire before taking part in a 7-day daily diary study. Measures of daily stress, perfectionistic thinking and disordered eating attitudes and behaviours were completed daily before bed. Results: Analyses found significant effects of childhood trauma on daily disordered relationship with food, concerns over food and weight gain and restrictive and compensatory practices. Higher levels of perceived daily stress were associated with higher scores on disordered relationship with food and concerns over food and weight gain, with childhood trauma found to moderate the relationship between daily stress and restrictive and compensatory practices. Daily perfectionistic thinking was only associated with higher daily concerns over food and weight gain. Childhood trauma did not moderate any of the perfectionistic thinking-eating relationships. Conclusions: These findings highlight the impact of childhood trauma on disordered eating at the daily level and have implications for future research and clinical practice.

**Supplementary Information:**

The online version contains supplementary material available at https://doi.org/10.1007/s40653-026-00834-1.

## Introduction

### Childhood Trauma and Eating

The experience of childhood trauma has been consistently linked to a number of adverse health outcomes and behaviours in adolescence and adulthood, impacting both physical health and psychological wellbeing (O’Connor et al., 2021; Petruccelli et al., 2019; Rogerson et al., 2024). One outcome of interest is that of eating attitudes and behaviours, with childhood trauma linked to the onset, trajectory, and symptomology of diagnosed eating disorders. The overall prevalence of childhood trauma has been reported as two to four times higher amongst individuals with a diagnosed eating disorder in comparison to healthy controls (Molendijk et al., 2017). Furthermore, individuals with an eating disorder diagnosis have reported high rates of childhood sexual abuse, neglect, and physical abuse (Caslini et al., 2016; Pignatelli et al., 2015; Smolak & Murnen, 2002).

It has been suggested that a lack of emotional validation in childhood may result in the emergence of problematic and disordered eating symptomology, which develops as a coping strategy in order to manage overwhelming feelings, ultimately leading to clinical diagnosis for some (Pignatelli et al., 2015). Elsewhere, it has been proposed that childhood abuse negatively impacts the perception of body image, leading to eating disorder symptoms to overcome such dissatisfaction (Caslini et al., 2016). Indeed, research has found that individuals with a diagnosed eating disorder who have also experienced childhood trauma score significantly higher than non-trauma groups on measures of food restriction, concerns over weight and shape, and reduced daily functioning (Guillaume et al., 2016). Additionally, it has been found that individuals with experiences of childhood trauma had an earlier age of eating disorder onset, a higher frequency of bingeing and purging episodes, and increased usage of laxatives or diuretics to control weight (in comparison to a non-trauma group; Molendijk et al., 2017).

However, it is not only diagnosed eating disorders that are of importance when examining the relationship between childhood trauma and eating. It has been proposed that diagnosable eating disorders lie at the uppermost end of a continuum, documenting a progression from those considered normal or healthy eaters, through to those exhibiting disordered eating behaviours and attitudes (Scarano & Kalodner-Martin, 1994). It has been found that individuals at various points on the proposed continuum differ in regard to their eating behaviours, related cognitions (to food, body image, and weight), and perception of self (Scarano & Kalodner-Martin, 1994).

Disordered eating outcomes encompass both eating-related attitudes and behaviours. Attitudes may relate to the ideas, beliefs and assumptions that impact our relationship with food and influence the choices we make about what, how much, and when we eat; disordered attitudes may include excessive preoccupation with such (Alvarenga et al., 2010a) Disordered eating behaviours may include bingeing, purging, laxative or diuretic abuse, and restrictive dieting by food avoidance or calorie restriction (Emery et al., 2021). Indeed, childhood trauma has been linked to disordered eating outcomes amongst non-clinical populations, without an eating disorder diagnosis (Emery et al., 2021; Fuemmeler et al., 2009; Hasselle et al., 2017; Smyth et al., 2008; Yoon et al., 2022).

The experience of severe trauma in childhood has been found to significantly correlate with restricted eating behaviours (Smyth et al., 2008) and greater self-reported disordered eating in adulthood (Hasselle et al., 2017). Considering specific attitudes and behaviours, childhood trauma has been associated with a greater risk of overeating, binge eating, unhealthy weight control behaviours, chronic dieting, and weight and shape concerns (Emery et al., 2021) Some gender and trauma specific outcomes have been identified, such as findings that the highest risk ratios were for physical neglect and binge eating (amongst females) and emotional abuse and unhealthy weight control (amongst males) (Yoon et al., 2022). Furthermore, it has been found that women with a history of childhood physical abuse were more likely to report skipping meals in order to lose weight, and those with histories of physical or sexual abuse were more likely to report a fear of losing control when eating (Fuemmeler et al., 2009).

However, previous research into the link between childhood trauma and non-clinical disordered eating outcomes has often utilised measures originally designed to capture eating disorder symptomology, such as the SCOFF questionnaire (Morgan, 1999) or the Eating Disorder Examination Questionnaire (Fairburn & Begin, 1993). Others have captured eating attitudes and behaviours using limited items (Fuemmeler et al., 2009; Emery et al., 2021). Moreover, there is a lack of research utilising measures designed to capture non-clinical outcomes, making this a key aim of the current study.

### The Role of Stress and Perfectionistic Thinking

Stress has been examined in relation to eating behaviours, with research finding variability in both the amounts and types of food consumed when experiencing stress (O’Connor & Conner, 2011; Hill et al., 2022; Torres & Nowson, 2007). Under conditions of stress, between-person differences are evident, whereby some individuals report increasing food consumption, others eat much less than usual, and a smaller minority do not significantly alter their habits (Yau & Potenza, 2013). Most research into stress and eating has focused on the amount and type of food consumed, such as overall calorie intake, healthy versus unhealthy food, and between-meal snacking (O’Connor & Conner, 2011). However, it remains less clear what the impact of stressors are on eating attitudes as well as on behaviours. Moreover, research has also been extremely limited in considering the role childhood trauma may play in understanding the relationship between stress and eating attitudes and behaviours. Previous work has found significant associations between childhood trauma and stress-related eating captured by binge eating and coping-motivated eating (Mason et al., 2015). However, few studies have investigated childhood trauma, daily stress and (disordered) eating attitudes and behaviours simultaneously in the same study.

A further area of interest in relation to childhood trauma and eating attitudes and behaviours is perfectionistic thinking. Whilst perfectionism has been implicated as a “necessary condition” for the development of later eating disorders (Goldner et al., 2002), and elevated perfectionism traits have been linked to experiences of early trauma (Chen et al., 2019), little research has examined these together. Additionally, perfectionistic thinking has received minimal attention within the literature, outside of research linking greater perfectionistic thinking to bulimia related cognitions (Flett et al., 2011), or anorexic and bulimic behaviours (Downey et al., 2014). Therefore, a further key aim of the current study was to examine the role of stress and perfectionistic thinking in the childhood trauma and disordered eating relationship.

### Daily Diary Approaches

One of the limitations of many studies that have sought to investigate the relationship between childhood trauma and eating outcomes relates to the study designs. Typically, studies have been retrospective and observational in nature (Petruccelli et al., 2019), or cross-sectional and laboratory-based (O’Connor & Conner, 2011). Daily diary designs instead allow for the measurement of both within and between-person variables, allow for the study of “natural history” patterns, and are appropriate for usage to capture micro-level processes and state-like fluctuations in variables of interest (Conner & Lehman, 2012; Gunthert & Wenze, 2011; O’Connor & Ferguson, 2008). Previous daily diary studies have found associations between daily hassles (stress) and increased between-meal snacking (Moss et al., 2021; O’Connor et al., 2008), daily stress and an increased likelihood of eating to cope with difficult emotions (Hsu & Raposa, 2020) and daily increases in perfectionistic thinking and an increased desire for thinness and higher levels of binge eating (Boone et al., 2012). However, to date no studies have sought to examine the impact of daily stressors and daily perfectionistic thinking on a wider range of eating attitudes and behaviours, whilst also exploring the direct and moderating effects of childhood trauma.

Therefore, taken together, the present study aimed to examine the following research questions:

1. Is there a main effect of childhood trauma on daily eating outcomes, whereby childhood trauma directly influences daily eating attitudes and behaviours in adulthood?
2. Are more maladaptive or disordered eating attitudes and behaviours evident on days with [a] more stressors, and [b] greater perfectionistic thinking?
3. Are the above relationships (in #2) moderated by childhood trauma, such that the effects of daily stress and perfectionistic thinking on eating attitudes and behaviours are stronger in those who have experienced high levels of childhood trauma (in comparison to those with lower levels or no experience of childhood trauma)?

## Method

### Design and Participants

A multi-level, prospective, interval-contingent daily diary design was utilised, whereby participants completed an online diary once per day, in the evening (at the end of the day), for seven consecutive days. The study received ethical approval from the University of Leeds School of Psychology ethics panel (reference number PSYC-454). Participants were recruited from the community using a variety of online platforms, including social media, online participant recruitment websites, and research recruitment forums and pages. All participants were required to be 18 years old or above, and able to read English fluently. Participants that provided at least 3 days of diary data were included in the analyses (see below). As a result, 105 participants with a total of 674 diary days were included. A summary-statistics-based power analysis to detect cross-level effects (Murayama et al., 2022) using data from a pilot study confirmed that a sample size of at least 93 participants would be sufficient to achieve 80% power (t = 1.58, df = 28). The study was pre-registered on AsPredicted (https://aspredicted.org/vb8xg.pdf) on the 29^th of^ July 2022.

### Procedure

Prospective participants were invited to participate in a study investigating the relationship between childhood experiences and eating. Participants firstly completed an online background questionnaire and then signed up to receive a weblink to their daily diary. Text messages containing links to each daily diary were sent for seven consecutive days. Following completion of the daily diaries, participants were debriefed.

### Background Questionnaire Measures

The background questionnaire collected demographic information (age, sex, ethnicity, etc.) and included assessments of childhood trauma and eating attitudes and behaviours.

### Childhood Trauma

Childhood trauma was measured using the Childhood Trauma Questionnaire-Short Form (CTQ-SF; Bernstein et al., 2003), a 28-item, self-report measure that captures five categories of childhood trauma (emotional abuse, physical abuse, sexual abuse, physical neglect, and emotional neglect). Respondents are asked to retrospectively indicate whether they have experienced a number of occurrences during childhood, with responses measured on a five-point Likert scale from 1 (never true) to 5 (often true). Higher scores are reflective of a higher incidence and severity of abuse. The CTQ-SF has demonstrated good criterion-related validity, excellent internal consistency, structural validity, and cross-cultural validity (Bernstein et al., 2003; Georgieva et al., 2021; Saini et al., 2019).

### Daily Questionnaire Measures

The daily questionnaire was designed to measure daily experiences of stress, perfectionistic thinking, and eating outcomes.

### Daily Stress

Participants were firstly asked to rate how stressed they felt today on a five-point Likert scale from not at all stressed to extremely stressed. In addition, the PSS-4 (Cohen et al., 1983) was adapted to the daily level by asking participants how often they felt or thought a certain way today (as opposed to the past month, e.g., today, how often did you feel that things were going your way?). Each item was rated on a five-point Likert scale from never to very often.

### Daily Perfectionistic Thinking

The Perfectionistic Cognitions Inventory was adapted for usage at the daily level. The five highest loading items from the original PCI (Flett et al., 1998) were adapted to reflect cognitions that had occurred that day, as opposed to within the previous week. The two highest loading statements were deemed inappropriate due to the usage of the word “work”, which may not have been applicable to some participants due to current employment circumstances or perception of work. Therefore, the third to seventh highest loading items were adapted. For example, “I expect to be perfect” was adapted to “I expected to be perfect”. Items were rated on a five-point Likert scale from 0 (not at all) to 4 (all of the time).

### Daily Eating Attitudes and Behaviour

To measure disordered eating attitudes and behaviours, 10 items from Part II of the Disordered Eating Attitude Scale, English version (DEAS; Alvarenga et al., 2010a) were adapted to reflect daily occurrences of such (see supplementary materials for full description of the DEAS). The top two loading items from each of the five subscales were adapted: relationship with food, concerns about food and weight gain, restrictive and compensatory practices, feeling toward eating, and idea of normal eating. For one subscale (feeling toward eating), the highest loading item did not appropriately translate to the daily level, and therefore the second and third highest loading items were used instead.

### Analysis

Multilevel analysis was conducted using HLM 8.2 software. Data was excluded from analysis if less than three days of diaries were completed. The data contained a two-level hierarchical structure. Level 1 represented the within-person variation (daily stress, daily perfectionistic thinking, and daily eating attitudes and behaviours) and Level 2 represented the between-subject variation (childhood trauma). Level 1 variables were centred around the group mean and Level 2 variables were grand mean centred. The Level 1 slope (models) were examined to investigate the relationship between either daily stress or daily perfectionistic thinking, and daily eating attitudes and behaviours. Level 2 slopes (models) were also examined to test whether these relationships were moderated by childhood trauma. Therefore, to test cross-level interactions, either (1) perceived daily stress (level 1 variable) was added as the predictor for each daily eating attitudes and behaviour outcome (level 1 variables), with childhood trauma (level 2 variable) added as a moderator, or (2) daily perfectionistic thinking (level 1 variable) was added as the predictor for each daily eating attitudes and behaviour outcome (level 1 variables), with childhood trauma (level 2 variable) added as a moderator. Any significant cross-level interactions where childhood trauma was found to moderate a stress-eating outcome or perfectionistic thinking-eating outcome, were examined using Preacher’s simple slopes analysis for cross-level interactions (Preacher et al., 2006). Note some additional analyses were conducted on between-meal snacking and are reported in the supplementary materials.

## Results

### Participant Demographics

One hundred and five participants were included in the analysis with an average age of 35.03 years old (*SD =* 13.24, range 18–72). The majority identified as female (*n =* 84, 80%) and most participants described their ethnic background as White (*n =* 77, 73.77%) and their ethnicity as British (*n =* 33) or English (*n =* 44). Additionally, most participants had been educated to undergraduate (*n* = 33, 31.43%) or postgraduate-degree level (*n* = 39, 37.14%). Most participants were categorised as having a healthy BMI (*n =* 43) according to NHS criteria (NHS, 2023). Table 1 displays the descriptive statistics for Level 1 (within-person) and Level 2 (between-person) variables.

**Table 1:** Descriptive statistics for level 1 and level 2 study variables (*n =* 105)

	Mean	SD	Range
**Level 1 variables**			
Daily perceived stress	6.10	3.66	0–16
Daily perfectionistic thinking	5.91	5.47	0–20
Daily DEAS			
Relationship with food	2.64	1.49	2–10
Concerns about food and weight gain	3.15	1.95	2–10
Restrictive and compensatory practices	2.84	1.91	2–10
Feeling toward eating	4.65	1.60	2–10
Idea of normal eating	3.86	2.15	2–10
**Level 2 variables**			
Childhood trauma			
Emotional abuse	11.75	6.47	5–25
Physical abuse	7.56	4.18	5–23
Sexual abuse	7.89	5.67	5–25
Emotional neglect	11.91	5.61	5–25
Physical neglect	8.49	4.07	5–22
Total	47.6	20.36	25–108

DEAS = Disordered Eating Attitudes Scale

**Table 2:** Effects of Total childhood trauma on daily DEAS subscales

HLM effect	Symbol	Coeff	SE	*p*
*Intercept*				
CTQ - daily relationship with food	*β*_*01*_	0.032	0.006	< 0.001
CTQ - concerns about food and weight gain	*β*_*01*_	0.032	0.008	< 0.001
CTQ - restrictive and compensatory practices	*β*_*01*_	0.036	0.007	< 0.001
CTQ - feeling toward eating	*β*_*01*_	0.011	0.006	0.073
CTQ - idea of normal eating	*β*_*01*_	0.016	0.010	0.117

CTQ = total Childhood Trauma Questionnaire score, DEAS = Disordered Eating Attitude Scale

### Main Effect of Childhood Trauma on Daily DEAS Subscales

**Fig. 1:**
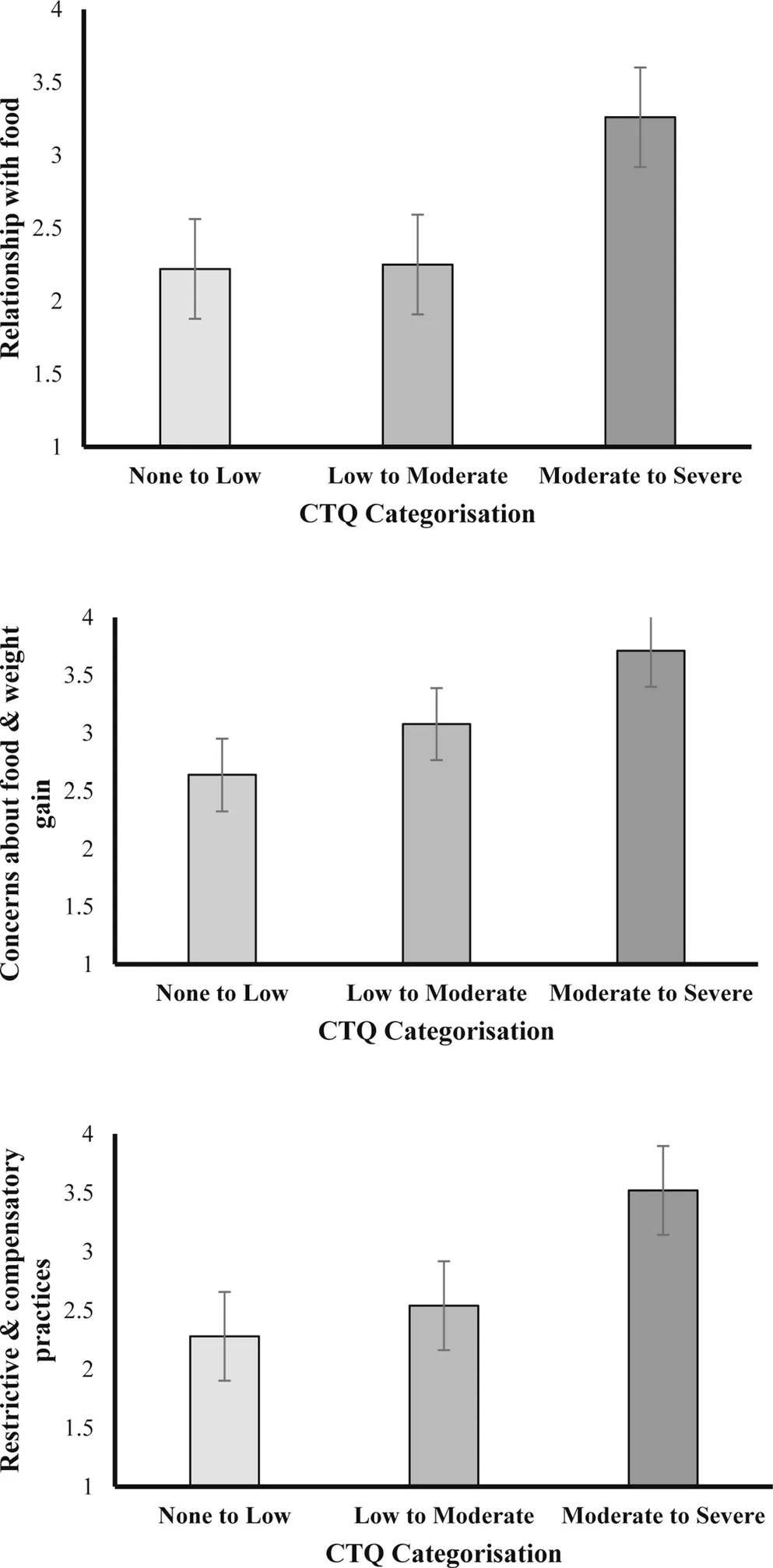
Effects of childhood trauma on mean daily Disordered Eating Attitude Subscale scores (error bars represent standard error of the mean)

A significant main effect of childhood trauma was found for three of the five daily DEAS subscales, as reported in Table 2. Firstly, there was a significant main effect of total CTQ on the DEAS relationship with food subscale (*β*_*01*_ = 0.032, *p = <* 0.001), indicating that higher levels of childhood trauma were associated with a higher daily disordered relationship with food. Figure 1 (upper panel) shows that the mean relationship with daily food subscale was higher in participants categorised as scoring “Moderate to Severe” on the CTQ than participants categorised as scoring “None to Low” and “Low to Moderate”. Similarly, a significant main effect was also found for the total CTQ score on DEAS concerns about food and weight gain subscale (*β*_*01*_ = 0.032, *p = <* 0.001) and the DEAS restrictive and compensatory practices subscale (*β*_*01*_ = 0.036, *p = <* 0.001), indicating that higher levels of childhood trauma were associated with greater daily concerns about food and weight gain and restrictive and compensatory practices (see Fig. 1, middle & lower panels, respectively).

### Daily Stress, Daily Eating Measures, and Childhood Trauma Moderation

Next HLM analyses were also used to investigate the relationship between the daily stress and daily eating measures, and whether these relationships were moderated by childhood trauma (Table 3, upper panel). Daily stress was positively associated with the daily relationship with food (*β*_*10*_ = 0.039, *p =* 0.007) and with daily concerns about food and weight gain (*β*_*10 =*_ 0.039, *p =* 0.039. These relationships were not moderated by childhood trauma.

**Table 3:** Effects of daily stress (upper panel) and daily perfectionistic thinking (lower panel) on daily eating attitudes and behaviours and whether these relationships are moderated by childhood trauma

Model and Variables	β	Coeff	SE	*p*
***Daily PSS, DEAS outcomes and CTQ Total***				
Relationship with food				
PSS – daily relationship with food	*β*_*10*_	0.039	0.014	0.007
CTQ x PSS – daily relationship with food	*β*_*11*_	0.001	0.0009	0.271
Concerns about food and weight gain				
PSS – concerns about food and weight gain	*β*_*10*_	0.039	0.018	0.039
CTQ x PSS – concerns about food and weight gain	*β*_*11*_	0.002	0.001	0.063
Restrictive and compensatory practices				
PSS – restrictive & compensatory practices	*β*_*10*_	0.013	0.015	0.376
CTQ x PSS – restrictive & compensatory practices	*β*_*11*_	0.001	0.0007	0.038
Feeling toward eating				
PSS – feeling toward eating	*β*_*10*_	−0.039	0.023	0.089
CTQ x PSS – feeling toward eating	*β*_*11*_	−0.002	0.001	0.835
Idea of normal eating				
PSS– idea of normal eating	*β*_*10*_	0.057	0.029	0.056
CTQ x PSS – idea of normal eating	*β*_*11*_	−0.009	0.001	0.508
***Daily PCI***,*** DEAS outcomes and CTQ Total***				
Relationship with food				
PCI – daily relationship with food	*β*_*10*_	0.014	0.009	0.114
CTQ x PCI – daily relationship with food	*β*_*11*_	0.0001	0.0007	0.856
Concerns about food and weight gain				
PCI – concerns about food and weight gain	*β*_*10*_	0.029	0.014	0.039
CTQ x PCI – concerns about food and weight gain	*β*_*11*_	−0.005	−0.473	0.637
Restrictive and compensatory practices				
PCI – restrictive & compensatory practices	*β*_*10*_	0.013	0.016	0.428
CTQ x restrictive & compensatory practices	*β*_*11*_	0.005	0.001	0.613
Feeling toward eating				
PCI – feeling toward eating	*β*_*10*_	−0.021	0.017	0.224
CTQ x PCI – feeling toward eating	*β*_*11*_	−0.009	0.0009	0.268
Idea of normal eating				
PCI – idea of normal eating	*β*_*10*_	0.033	0.019	0.083
CTQ x PCI – idea of normal eating	*β*_*11*_	−0.005	0.001	0.625

PSS = daily perceived stress, PCI = daily perfectionistic thinking, CTQ = total ChildhoodTrauma Questionnaire, DEAS = Disordered Eating Attitudes Scale

**Fig. 2:**
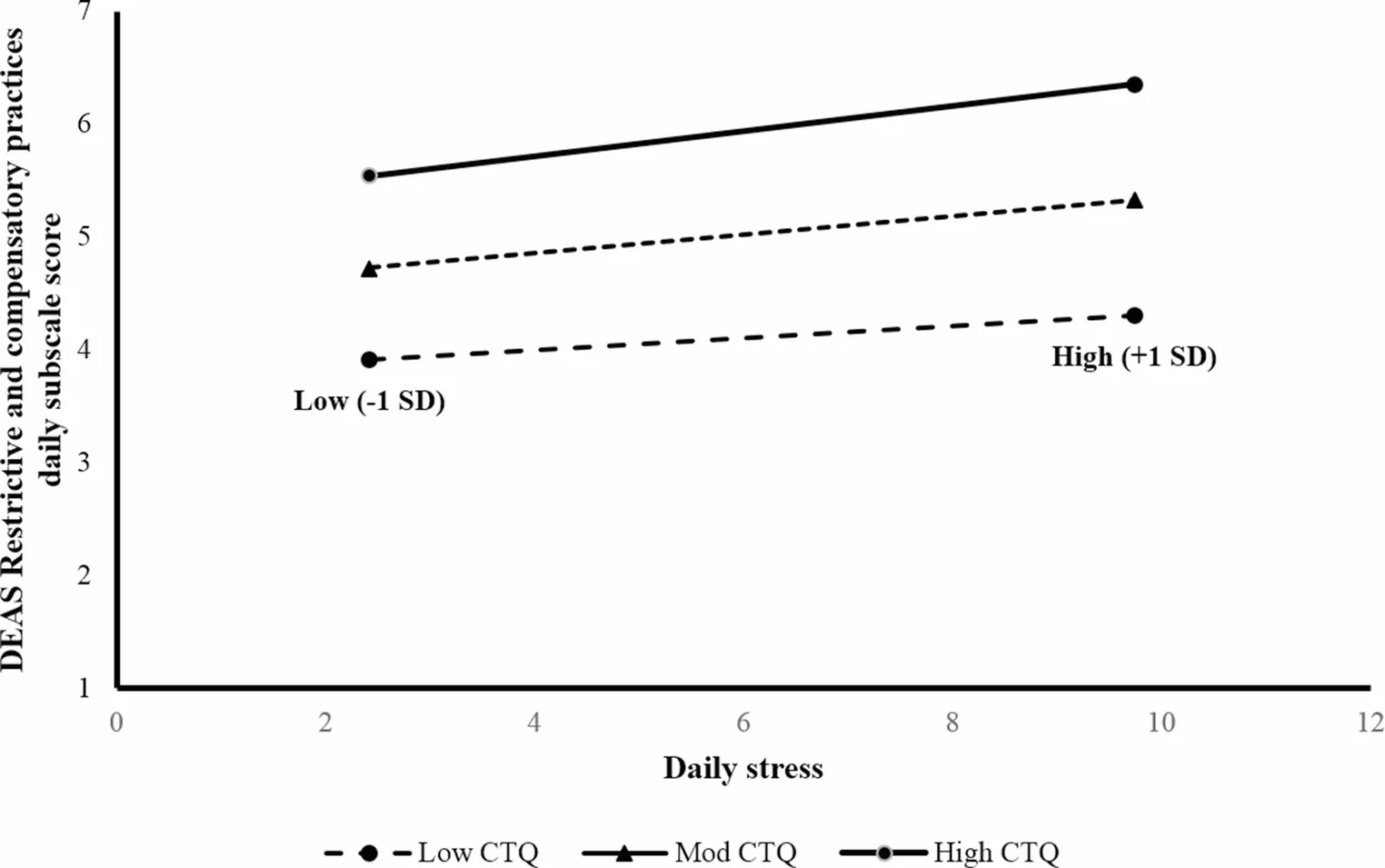
The relationship between daily perceived stress and daily restrictive/compensatory practices at different levels of childhood trauma

Daily stress was not significantly associated with restrictive and compensatory practices (*β*_*10*_ = 0.013, *p =* 0.376). However, CTQ was found to moderate this relationship (*β*_*11 =*_ 0.001, *p =* 0.038). This interaction was decomposed using simple slopes following the steps outlined by Preacher et al. (2006) These analyses showed that the impact of daily stress on restrictive practices was stronger in individuals with higher compared to lower levels of childhood trauma. Daily stress was significantly related to greater restrictive and compensatory practices at low (*β =* 0.053, *p =* 0.043), moderate (*β =* 0.083, *p =* 0.033), and high (*β =* 0.110, *p =* 0.03) levels of childhood trauma with the strongest associations found at high levels. Daily stress was not significantly associated with feeling toward eating (*β*_*10*_ = −0.038, *p =* 0.089), or idea of normal eating (*β*_*10*_ = 0.056, *p =* 0.056) and CTQ did not moderate either of these relationships (Fig. 2).

### Daily Perfectionistic Thinking, Eating Outcomes, and Childhood Trauma Moderation

Finally, HLM analyses were used to investigate the relationship between the daily perfectionistic thinking, daily eating outcomes, and the moderation effect of childhood trauma (Table 3, lower panel). Daily perfectionistic thinking was positively and significantly associated with daily concerns about food and weight gain (*β*_*10*_ = 0.029, *p =* 0.039). This indicated that, on days with higher levels of perfectionistic thinking, participants reported higher scores relating to concerns over food and weight gain. Daily perfectionistic thinking was not significantly associated with relationship with food, restrictive and compensatory practices, feeling toward eating, or idea of normal eating. Childhood trauma did not moderate any of the daily perfectionistic thinking and eating measure relationships (see Table 3).

## Discussion

This study aimed to examine the impact of childhood trauma on daily eating attitudes and behaviours, and the impact of daily experiences of stress and perfectionistic thinking within this relationship. The main findings were that childhood trauma was significantly associated with a greater daily disordered relationship with food, higher concerns about food and weight gain, and increased restrictive and compensatory practices in adulthood. Furthermore, days with greater perceived stress were associated with having a higher disordered relationship with food and concerns about food and weight gain, and greater daily perfectionistic thinking was associated with increased concerns about food and weight gain.

These findings are important as they show that trauma in childhood can influence eating attitudes and behaviours in adulthood that may increase the risk of developing an eating disorder. For example, it has been found that individuals at greater risk of developing an eating disorder, score higher on the DEAS, indicating greater disordered eating attitudes and behaviours (Alvarenga et al., 2015). This suggests that it might be useful to deliver the DEAS amongst populations who have experienced childhood trauma to identify those who may be at risk of developing an eating disorder in the future.

A number of the present findings are also consistent with previous literature, such as greater childhood trauma being linked to an increased fear of losing control when eating (as measured by the relationship with food subscale) (Fuemmeler et al., 2009), greater concern with calorie intake and weight gain (Emery et al., 2021), and an increase in restriction or compensation such as skipping meals and engaging in unhealthy weight control behaviours (Smyth et al., 2008; Yoon et al., 2022). However, the current study may be deemed a valuable extension as it has demonstrated for the first time that these relationships are evident and observable at the daily level and not limited to one-off, snapshot retrospective assessment.

In the current study, participants with higher levels of childhood trauma did not report daily increases in eating to manage difficult emotions such as being sad, bored, or angry (captured within the idea of normal eating subscale) which contrasts with previous findings (e.g., Mason et al., 2015) and the proposed explanations of disordered eating that suggest such behaviours are used to manage overwhelming or distressing feelings amongst those who have been invalidated in childhood (Pignatelli et al., 2015). However, previous research has also suggested that the belief of such emotions is important, with disordered eating more likely to occur in individuals who view difficult emotions as uncontrollable (Strodl & Wylie, 2020). It may be that for participants in the current study, experiencing sadness, boredom, or anger did not result in reports of disordered eating as the emotions were not perceived as uncontrollable or intense. The impact of emotion regulation is one of the most consistently researched in the childhood trauma and disordered eating outcomes literature (Rabito-Alćon et al., 2021); extending such research to the daily level may provide a more detailed explanation of the present finding.

The current study also found that childhood trauma impacts feeling toward eating, calorie and weight concerns, and restrictive and compensatory practices. These findings may be explained in a number of different ways. One such explanation is that individuals with experiences of trauma in childhood have higher levels of body dissatisfaction and negative perceptions of body image, and therefore engage in food restriction and compensation to manage these issues (Caslini et al., 2016). Indeed, childhood trauma has been found to significantly affect an individual’s perception of their body image and related cognitions such as disgust and shame (Bödicker et al., 2022), and can result in greater disordered eating such as restrictive and compensatory practices (Vartanian et al., 2018). Whilst the perception of body image was not measured in the current study, participants with higher levels of childhood trauma did indeed demonstrate greater food and weight concerns which may have subsequently impacted what they ate.

It has been proposed that individuals with histories of abuse experience a greater perceived loss of control over general life circumstances, that may also extend to eating (Polivy & Herman, 2002); regaining control of eating may be a way of attaining pride and self-management when other aspects of life feel most uncontrollable. This may explain why individuals in the present study were also significantly more likely to skip meals or only consume liquids. A fear of losing control when eating is often associated with binge eating disorder or episodes of binge eating (Molendijk et al., 2017). Scarano and Kalodner-Martin’s (1994) continuum of eating states a fear of losing control to be a “potent” factor in understanding differences between those with and without an eating disorder diagnosis who engage in binge eating behaviours. Whilst daily binge eating was not captured in the current study, the novel finding here that individuals with higher levels of childhood trauma reported greater daily occurrences of eating less in front of others and to overeat when alone may be of importance, as such practices have been reported amongst individuals who binge eat (Westerberg & Waitz, 2013).

The finding that childhood trauma was associated with a greater disordered relationship with food, reflecting higher feelings of shame, anger or guilt related to food are consistent with previous research whereby abuse in childhood has been linked to later body-related shame and eating difficulties (Gilbert & Thompson, 2002). It has been found that an absence of caring, soothing early life experiences may result in increased self-criticism and body-oriented shame, subsequently leading to disordered eating (Gois et al., 2018). The current findings may therefore be partially explained by the role and experience of shame, resulting in greater awareness of what participants ate as well as attempts to manage such (via food restriction or compensation). Future research ought to investigate these potential mechanisms further.

We also found that higher levels of daily perceived stress were significantly associated with increases in a daily disordered relationship with food and concerns about food and weight gain, extending previous research that has examined the impact of stress on amount and type of food consumed (O’Connor et al., 2008; Debeuf et al., 2018). Whilst previous research has found increased perceived stress results in a greater fear of losing control when eating (Groesz et al., 2012), the present study also found this to occur at the daily level. It may be that participant awareness of a tendency to rely on food during stressful days resulted in a greater fear of the likelihood of such happening, regardless of whether this subsequently occurred. Furthermore, the broad definition of stress refers to a generalised sense of worry and reduction in perceived ability to cope; hence, a loss of control (Fink, 2010; Manosso et al., 2022). Therefore, it may be that a fear of losing control when eating at the daily level is an extension of more generalised worries or anxieties in the context of increased stressors, which may also relate to the present finding regarding increased stress and concerns over weight gain and calorie intake.

The current study also found that on days with greater perfectionistic thinking, participants expressed a greater concern over food and weight gain, but this did not significantly impact the other four disordered eating subscales. These findings are perhaps somewhat unexpected for a number of reasons, not least because of the key role perfectionism has been shown to play in the development of disordered eating (Bardon-Cone et al., 2007; Goldner et al., 2002; Vacca et al., 2021). In particular, the finding that perfectionistic thinking did not lead to greater daily incidence of restrictive or compensatory practices is surprising, given that previous research has shown this to be an important relationship (Downey et al., 2014; Flett et al., 2011).

It may be that perfectionism is more likely to influence eating outcomes amongst individuals with the most disordered eating behaviours or those with clinical diagnoses, as per previous research (Downey et al., 2014). In the present study, whilst the average score on the background measure of the DEAS was higher than previously reported norms (Alvarenga et al., 2010b), outcomes at the daily level showed fewer participants scoring toward the upper range. Furthermore, although perfectionistic cognitions account for variability in state, as opposed to trait, perfectionism, it may be that individuals are less likely to demonstrate daily fluctuations in perfectionistic thinking and therefore these have less of an impact on eating outcomes than multi-dimensional trait perfectionism (Boone et al., 2012).

The current findings highlight the importance of recognising that experiences of childhood trauma may increase the risk of individuals engaging in disordered eating outcomes that may not reach the threshold for clinical diagnoses but may still be damaging for health. Furthermore, control has been widely reported within the eating outcomes literature, however, over-emphasising the role of personal control can risk increasing blaming or stigmatised attitudes toward individuals who struggle with eating (Branley-Bell et al., 2023). The current findings also draw attention to the links between childhood trauma, shame, and subsequent eating outcomes, and therefore within clinical practice, compassion-focused interventions may be helpful to reduce self-criticism and increase body satisfaction to reduce disordered eating outcomes (Albertson et al., 2014). Furthermore, the finding that individuals in the present study were not more likely to eat more in response to difficult emotions and in the context of increased daily stress may highlight the role of health promotion via other methods of coping with stress, such as exercise to increase positive affect (Thome & Espelage, 2004). The current findings continue to underscore the importance of examination of non-clinical eating attitudes and behaviours, and subsequent targeted interventions to identify those who may be at risk of later developing more pathological eating outcomes or reach levels of clinical diagnosis (Scaranao & Kalodner-Martin, 1994).

Finally, the importance of examining variables at the daily level is further emphasised by the current findings, given the opportunity to capture subtle alterations in disordered eating attitudes and behaviours alongside influential factors such as perceived stress or perfectionistic thinking. Identifying nuanced changes in cognitions and behaviours at the daily level could serve to more readily inform preventative strategies, for example identifying those whom may be at risk to developing greater disordered eating outcomes and allowing opportunities for earlier intervention as part of wider prevention efforts (Neumark-Stztainer et al., 2006; Scarano & Kalodner-Martin, 1994).

Whilst the study aimed to recruit a diverse sample, most participants identified as female, White, and of British nationality. Therefore, the generalisability of findings to other cultures or ethnicities may be limited. Indeed, differences relating to eating attitudes and beliefs and indeed exposure to daily stressors may be influenced by cultural or familial ideals relating to body image, perceived discrimination, or media exposure (e.g., Cheng et al., 2018; Cuevas et al., 2019; Weiss, 1995).

Furthermore, the sample size was relatively small and therefore future research should focus on replication of the findings amongst a larger sample. It is acknowledged that the measurement of trauma (CTQ-SF) is a retrospective, self-report tool that may be subject to a degree of response bias or repression and experiences cannot be independently validated. However, this point notwithstanding, it has been suggested that tools such as the CTQ may represent under-reporting of actual abuse or neglect occurrence (Hardt & Rutter, 2004).

## Conclusions

The current findings highlight the importance of childhood trauma in understanding the development of, and impact on, disordered eating attitudes and behaviours at the daily level in adulthood. They have important implications for how health professionals may formulate difficulties relating to disordered eating behaviours through exploration of childhood trauma experiences, which influences later intervention. The role of stress and perfectionistic thinking within this relationship warrants further exploration, and future research should seek to further investigate the moderating role of childhood trauma in the relationship between daily variables and daily disordered eating outcomes.

## Supplementary Information

Below is the link to the electronic supplementary material.

Supplementary Material 1 (DOCX 15.2 KB)

## Data Availability

Data available upon reasonable request.
