# Supplementary material for: Childhood Trauma and Disordered Eating Attitudes and Behaviours in Adulthood: Investigating the Role of Daily Stress and Daily Perfectionistic Thinking
Source: J Child Adolesc Trauma. 2026 Feb 25;19(3):1141–52. doi: 10.1007/s40653-026-00834-1 (PMC13546233; doi:10.1007/s40653-026-00834-1)
Supplement: Supplementary file 1 [file 40653_2026_834_MOESM1_ESM.docx]

**Supplementary materials**

The Disordered Eating Attitude Scale (DEAS; Alvarenga et al., 2010a) is a two-part, 25-item scale designed to capture disordered eating attitudes, relationships, and beliefs toward food that may be evident without an eating disorder diagnosis. The scale comprises five subscales; relationship with food (e.g., I am afraid to start eating and not be able to stop), concerns about food and weight gain (e.g., I quit eating a kind of food if I find out it has more calories than I thought), restrictive and compensatory practices (e.g., do you skip meals to avoid putting on weight?), feeling toward eating (e.g., do you feel pleasure when you eat?), and idea of normal eating (e.g., do you believe it is normal to eat sometimes just because you are sad, upset, or bored?). In Part I, respondents answer questions relating to how healthy and necessary they consider the consumption of a range of food types to be (e.g., sugar, breads, fruit), from options of often, occasionally, or not, scored between 1 and 3 points. Part I also contains 10 closed questions with “Yes” or “No” responses, toward food and eating (scored as 1 or 5 points). The final question within Part I asks participants what their behaviour is after eating more than usual, with 5 responses scored between 1 and 5 points. Part II contains 13 questions rated on a five-point Likert scale from 1 (rarely/never) to 5 (always). Scores can be summed for each subscale as well as an overall total, with higher scores indicative of more disordered attitudes and behaviours toward food and eating. The reliability of the DEAS as measured in the original study was found to be good (*a* = 0.75) (Alvarenga et al., 2010b).

*Additional analyses – between-meal snacks and fruit/vegetable consumption*

Participants were also asked how many portions of fruit and vegetables they had eaten that day and to report any between-meal snacks they had consumed that day. Between-meal snacks were coded into total number of snacks, total healthy snacks, and total unhealthy snacks, categorised using food composition tables (McCance & Widdowson, 2014). A snack was considered unhealthy if it contained high levels of sugar and/or fat (more than 17.5g fat or 22.5g sugar per 100g of food).

The results showed that there was no significant main effect of childhood trauma on total daily snacks (*β_01_* = -0.003, p = 0.626), daily unhealthy snacks (*β_01_* = -0.001, p = 0.78), or daily healthy snacks (*β_01_* = -0.002, p = 0.678). Daily stress was positively and significantly associated with total daily healthy snacks (*β_10_* = 0.29, *p =* 0.036), suggesting that on days with greater stress, participants consumed a greater number of healthy snacks. There was no significant association between daily stress and daily total snacks (*β_10_* = 0.039, *p =* 0.058) or daily unhealthy snacks (*β_10_* = 0.008, *p =* 0.602). Daily perfectionistic thinking was significantly associated with daily unhealthy snacks (*β_10_* = 0.02, *p* = 0.035), suggesting that on days with greater perfectionistic thinking, participants consumed a greater number of unhealthy snacks. Daily perfectionistic thinking was not significantly associated with the number of total snacks consumed (*β_10_* = 0.027, *p =* 0.065) or daily healthy snacks (*β_10_* = 0.012, *p =* 0.207). Childhood trauma did not moderate any of the daily snack, daily stress, or daily perfectionistic thinking outcomes.

Additional analyses were conducted to determine whether there was a main effect of childhood trauma on fruit or vegetable consumption, whether daily stress and perfectionistic thinking influenced fruit or vegetable consumption, and whether these relationships were moderated by childhood trauma. There was no main effect for childhood trauma and fruit consumption (*β_01_* = -0.005, *p =* 0.22). A significant main effect was found for childhood trauma and vegetable consumption (*β_01 =_* -0.02, *p =* <0.001), suggesting that the higher the score on the childhood trauma scale, the lower the portions of vegetables consumed per day. The results showed that daily stress was not significantly associated with daily fruit (*β_10_* = -0.03, *p =* 0.065) or vegetable consumption (*β_10_* = 0.04, *p =* 0.097). Daily perfectionistic thinking was not significantly associated with daily fruit (*β_10 =_* 0.017, *p =* 0.20) or vegetable consumption (*β_10_* = 0.02, *p =* 0.40). Additionally, childhood trauma did not moderate any of these relationships.
